# IDEA-EXECUTION ABILITY, SELF-DETERMINATION, AND CHEERFULNESS PREDICT DEMENTIA ONSET OVER NINE YEARS

**DOI:** 10.1093/geroni/igad104.2410

**Published:** 2023-12-21

**Authors:** Yufang Tu, Teri Undem, Melissa O’Connor

**Affiliations:** North Dakota State University, Fargo, North Dakota, United States; North Dakota State University, Fargo, North Dakota, United States; North Dakota State University, Fargo, North Dakota, United States

## Abstract

The prevalence of dementia and its negative effect on well-being has made it a global public health concern. It is important to understand factors that predict the onset of dementia above and beyond age and physical health, to aid in preventative efforts. The current study investigated idea-execution ability, self-determination, and cheerfulness as predictors of the onset of dementia across a nine-year period. Longitudinal data from the first nine waves of the National Health and Aging Trends Study (NHATS) were used. NHATS participants are nationally representative of Medicare recipients in the United States. Data were initially collected in 2011 and annually thereafter. The current study included 6780 participants; 678 participants reported having a diagnosis of dementia at either baseline or a follow-up visit. Cox regression was used to examine the following self-report items as predictors of months-to-dementia onset: idea execution ability (“when I really want to do something, I usually find a way to do it”); self-determination (“other people determine most of what I can and cannot do”); and cheerfulness (“how often I feel cheerful”). Each predictor was time-invariant and scored on a 3-point scale so that higher scores were more positive. Covariates included baseline age group (five-year groups), sex (dichotomized), years of education, and self-rated overall health. Statistically significant results were shown for idea-execution (HR=0.79, p=0.008), self-determination (HR=0.69, p< 0.001), and cheerfulness (HR=0.91, p=0.047). Each of these potentially modifiable variables was associated with reduced odds of developing dementia, above and beyond demographic and health factors.

